# Non Alcoholic Fatty Liver Disease in Southern Iran: A Population Based Study

**DOI:** 10.5812/hepatmon.9248

**Published:** 2013-05-23

**Authors:** Kamran Bagheri Lankarani, Fariborz Ghaffarpasand, Mojtaba Mahmoodi, Mehrzad Lotfi, Nima Zamiri, Sayed Taghi Heydari, Mohammad Kazem Fallahzadeh, Najmeh Maharlouei, Meisam Babaeinejad, Soheila Mehravar, Bita Geramizadeh

**Affiliations:** 1 Health Policy Research Centre, Shiraz University of Medical Sciences, Shiraz, IR Iran; 2 Department of Radiology, Shiraz University of Medical Sciences, Shiraz, IR Iran; 3 Department of Biostatistics, Jahrom University of Medical Sciences, Jahrom, IR Iran; 4 Student Research Committee, Shiraz University of Medical Sciences, Shiraz, IR Iran; 5 Department of Pathology, Transplant Research Centre, Shiraz University of Medical Sciences, Shiraz, IR Iran

**Keywords:** Non alcoholic Fatty Liver Disease, Prevalence, Risk Factors, Metabolic Syndrome, Iran

## Abstract

**Background:**

Population based studies on prevalence and risk factors of NAFLD in Iranian population are few. The prevalence of NAFLD and non alcoholic steatohepatitis (NASH) in Iranians varies from 2.9% to 7.1% in general population and 55.8% in patients with type 2 diabetes mellitus.

**Objectives:**

To determine the prevalence and determinants of non alcoholic fatty liver disease (NAFLD) in a sample of adult Iranian general population.

**Patients and Methods:**

This was a cross-sectional study being performed in Shiraz, southern Iran during a 10-month period from November 2010 to September 2011 through cluster random sampling of Iranian general population in Shiraz region. All individuals undergone anthropometric, blood pressure measurements, thorough medical history and physical examinations. Laboratory measurements included fasting blood glucose (FBS), lipid profile, complete blood count (CBC) and liver function tests. NAFLD was diagnosed by transabdominal ultrasonography.

**Results:**

819 subjects were included in this study among which were 340 males (41.5%) and 479 females (58.5%) with the mean age of 43.1 ± 14.1 years. NAFLD was diagnosed in 176 (21.5%) subjects. Patients with NAFLD were significantly older (P < 0.001), had higher proportion of male gender (P = 0.004) and had higher BMI (P < 0.001). They also had higher prevalence of hypertension (P < 0.001), high FBS (P < 0.001), high cholesterol (P = 0.026), high triglyceride (P < 0.001) and high waist circumference (P < 0.001). Taking all these together, patients with NAFLD had significantly higher prevalence of metabolic syndrome when compared to healthy subjects (P < 0.001).

**Conclusion:**

The prevalence of NAFLD in this group of Iranian adult general population is 21.5%. NAFLD in Iranian population is associated with male gender, old age, obesity, and features of metabolic syndrome.

## 1. Background

Non alcoholic fatty liver disease (NAFLD) is defined as the deposition of lipid, especially triglyceride (5 – 10%), in hepatocytes exceeding 5% of total liver weight in the absence of other etiologies of hepatic damage including hepatitis viruses, alcohol consumption and metabolic diseases (1). The prevalence of the disease has increased dramatically during the previous decade probably because of both, the changes of life-style (decreased physical activity and alterations in dietary habits) and the increased detection rate (2). The prevalence of NAFLD in the general population varies according to the type of diagnostic tools used. It has been reported that 10–35% of general population suffers from NAFLD (2-4). Risk factors such as insulin resistance (IR), oxidative stress, diabetes, hyperlipidemia, obesity and metabolic syndrome play an important role in the pathogenesis of this disease(5-8).Metabolic syndrome and NAFLD shares similar prevalence pattern, pathogenesis, clinical features and outcome (9). NAFLD has also been associated with increased cardiovascular diseases risks factors including increased carotid artery wall thickness and lower endothelial flow-mediated vasodilation. Moreover, NAFLD is associated with greater overall mortality and independently predicts the risk of future CVD events (10).Interestingly, some studies showed that risk factors of NAFLD are correlated with socioeconomic determinants (11, 12). Santos and colleagues, in a community-based study showed that the prevalence of metabolic syndrome is significantly higher in women of lower socioeconomical classes as defined by income and education (12).The prevalence and associated risk factors of NAFLD may vary in different geographical region. Population based studies on prevalence and risk factors of NAFLD in Iranian population are few. The prevalence of NAFLD and non alcoholic steatohepatitis (NASH) in Iranians varies from 2.9% to 7.1% in general population (13-15) and 55.8% in patients with type 2 diabetes mellitus (16). NAFLD in Iranian children was associated with age, alanine aminotransferase (ALT), fasting insulin, total cholesterol, low density lipoprotein (LDL) cholesterol, triglyceride and IR (13).

## 2. Objectives

The main aim of this population-based study was to determine the prevalence of NAFLD and its risk factors in a sample of adult Iranian population of southern Iran.

## 3. Patients and Methods

### 3.1. Study Population

This was a cross-sectional study performed in Shiraz, southern Iran during a 10-month period from November 2010 to September 2011 including a randomly selected sample of general population living in Shiraz, the major metropolitan area in southern Iran. Shiraz is the capital and main city of Fars province with an estimated population of 1, 711, 186 according to the recent national census. Proportion weight-based random cluster sampling was used based on home address, postal zip codes and municipality regions. Participants were randomly selected from all 7 municipality regions of the Shiraz city.All inhabitants older than 18 years from the randomly selected addresses were invited to participate. Those who agreed to take part in the study had to call back and make an appointment with the study team for detailed history and physical examination and laboratory testing. We excluded pregnant women or those who had delivered within past six months. Non Iranians were also excluded. The study protocol was approved by Institutional Review Board (IRB) and the research ethics committee of Health Policy Research Center affiliated with Shiraz University of Medical Sciences. All the participants gave their informed written consents.

### 3.2. Study Protocol

All the participants were asked to attend the clinic after an overnight fasting. A team of two nurses and two physicians performed interviews, obtained medical histories and performed physical examinations. Physicians were gender identical for all participants. Standard questionnaires, designed by co-working of epidemiologists and hepatologists were used in this study. These questionnaires included demographic information (age, sex, place of residence, marital status, education, income and etc.), medical history and health relevant behaviours, i.e. alcohol consumption, smoking habits, dietary habits, physical activities and results of history taking and physical examinations. The anthropometric measurements were also performed by two nurses.Intravenous blood samples were drawn from each subject to measure fasting blood sugar (FBS), triglyceride (TG), cholesterol, low-density lipoprotein (LDL), high-density lipoprotein (HDL), thyroid stimulating hormone (TSH), serum albumin, serum transaminases (alanine aminotransferase and aspartate aminotransferase) and gamma-glutamyltranspeptidase (GGT). Complete blood count (CBC) was also performed for each individual. Body mass index was calculated by dividing weight in kilograms by height in meters squared. Overweight was defined as a BMI ≥ 25 kg/m^2^ in both male and female, according to the redefined WHO criteria in the Asia Pacific Region (17). Hypertension was diagnosed as a systolic blood pressure ≥ 140 mmHg or a diastolic blood pressure ≥ 90 mmHg, according to the WHO criteria. Hyperlipidemia was defined as a total cholesterol level ≥ 240 mg/dL or a triglyceride level ≥ 200 mg/dL. Fasting hyperglycemia was defined as fasting plasma glucose ≥ 126 mg/dL. ALT abnormalities were defined as ALT ≥ 55 IU/L for males and ≥ 38 IU/L for females. Diagnoses of diabetes mellitus were based on the WHO 1999 criteria (18). Participants who reported current use of anti-hypertension or anti-diabetes medications were regarded as having hypertension or diabetes, respectively.NAFLD was diagnosed by means of upper abdominal ultrasonography (US) based on increased echogenicity of the hepatic parenchyma with an attenuation of the portal vein or diaphragm echogenicity (19). Transabdominal ultrasonography was performed using a Shimadzu ultrasound machine (Shimadzu Inc., Tokyo, Japan) with a 5-MHz to 7-MHz transducer probe (curvilinear). All the ultrasonogrphic evaluations were performed by one experienced radiologist. The US diagnostic patterns of fatty liver disease based on the presence of a “bright” liver, with stronger echoes in the hepatic parenchyma than in the renal parenchyma, often associated with unusually fine liver texture and vessel blurring, in the absence of findings suggestive of other chronic liver diseases. The severity of fatty liver was classified into three degrees: grade 1, mild fatty liver, visualization of the diaphragm and the intra hepatic vessel borders. Grade 2, moderate fatty liver, echogenicity is moderately increased, with slightly impaired visualization of the diaphragm or intra hepatic vessels. Grade 3, severe fatty liver, echogenicity is markedly increased with poor or no visualization of the diaphragm, the intra hepatic vessels, and posterior portion of the right lobe (19).NAFLD was diagnosed based on sonography and absence of heart disease, acute or chronic liver disease, acute or chronic kidney disease, any malignancy, alcohol consumption [more than 40 g (male) or 20 g (female) of alcohol per day for over five years], pregnancy, liver masses, abnormal copper metabolism or thyroid function test and history of any medication with adverse effects on the liver. Only hepatitis B surface antigen-negative and hepatitis C antibody-negative patients were enrolled. We also calculated the NAFLD fibrosis score according to Angulo et al. (20). In order to determine those patient with significant risk of fibrosis the following formula was used: NAFLD fibrosis score = –1.675 + 0.037 × age (years) + 0.094 × BMI (kg/m^2^) + 1.13 × IFG/diabetes (yes=1, no=0) + 0.99 × AST/ALT ratio – 0.013 × platelet (×10^9^/l) ‒ 0.66 × albumin (g/dl). Values greater than 0.676 and lower than –1.455 were considered as the presence and absence of fibrosis, respectively. Values between these cut-offs were considered as intermediate risk.

### 3.3. Statistical Analysis

Statistical analyses were performed using the SPSS software, version 16.0 (SPSS Inc., Chicago, Ill., USA). The prevalence of NAFLD was calculated as a proportion of diagnosed patients to included subjects. The chi-square test was used to compare the proportions between those with NAFLD and normal population. Independent t-test was used for comparing the parametric data between two categories. The results are expressed as mean ± SD and proportions as appropriate. A two-tailed p-value less than 0.05 was considered statistically significant.

## 4. Results

Overall we included 819 subjects in this study among which were 340 males (41.5%) and 479 females (58.5%) with the mean age of 43.1 ± 14.1 (ranging from 18 to 88) years. The demographic and socioeconomic status of the participants is summarized in Table 1. Most of the participants (45.5%) were categorized as mid income while 288 (35.2%) patients where categorized as low income. Forty seven (6.3%) subjects were illiterate. Overall 649 (84.1%) subjects were married and 123 (15.9%) were single. Table 2 summarizes the anthropometric and biochemistry characteristics of the study population.

**Table 1.** Demographic and Socioeconomic Status of 819 Subjects Participating in the Study

Variable	Value (n=819)
**Age, y, (43.1 ± 14.1)**	
**18–29, No. (%)**	166 (20.3)
**30–39**	165 (20.1)
**40–49**	211 (25.8)
**50–59**	171 (20.9)
**≥60**	106 (12.9)
**Sex** **, No. (%)**	
**Male**	340 (41.5)
**Female**	479 (58.5)
**Income** **, No. (%)**	
**Low (< 400 USD/month)**	287 (35.0)
**Mid (400–1000 USD/month)**	483 (59.0)
**High (≥1000 USD/month)**	49(6.0)
**Years of Education** **, No. (%)**	
**Illiterate**	53 (6.4)
**< Diploma**	212 (25.9)
**Diploma**	285 (34.9)
**Bachelor of Science (BS)**	183 (22.3)
**Master of Science (MS)**	43 (5.2)
**Doctorate**	43 (5.2)
**Marital Status** **, No. (%)**	
**Married**	689 (84.1)
**Single**	130 (15.9)

**Table 2.** Anthropometric and Biochemical Characteristics of the 819 Participants

Variable	Value (n=819)
**BMI, kg/m^2^**	26.2 ± 4.5
Underweight, No. (%)	25 (3.1)
Normal, No. (%)	315 (38.5)
Overweight, No. (%)	322 (39.3)
Obese, No. (%)	128 (15.6)
Severe Obese, No. (%)	29 (3.5)
**Hypertension** **, No. (%)**	
Yes	168 (20.5)
No	651 (79.5)
**Fasting Blood glucose, mg/dL** **, No. (%)**	
High	167 (20.4)
Normal	652 (79.6)
**Cholesterol, mg/dL, No. (%)**	
High	88 (10.8)
Normal	731 (89.2)
**Triglyceride, mg/dL, No. (%)**	
High	287 (35.0)
Normal	532 (65.0)
**LDL, mg/dL, No. (%)**	
High	58 (7.0)
Normal	761 (93.0)
**HDL, mg/dL, No. (%)**	
Low	301 (36.7)
Normal	518 (63.3)
**Waist Circumference, cm, No. (%)**	
High	287 (35.0)
Normal	532 (65.0)
**Metabolic Syndrome, No. (%)**	
Yes	166 (20.3)
No	653 (79.7)

NAFLD was diagnosed in 176 (21.5%) subjects. Of these patients, 72 (8.8%) had concomitant rise of liver enzymes. Severe and moderate fatty liver disease was reported in 42 (5.1%) and 134 (16.4%) respectively, while 180 (22.0%) had mild disease. The mean calculated NAFLD fibrosis score was ‒2.541 ± 1.746 (range ‒32.709 to 2.199). According to this score, 640 (78.2%) had no fibrosis, 14 (1.7%) had significant fibrosis and 165 (20.2%) had intermediate fibrosis. We compared the socio-demographic variables as well as anthropometric and biochemistry measures between those with NAFLD and healthy subjects (Table 3).

Patients with NAFLD were significantly older (P < 0.001), had higher proportion of males (P = 0.004) and had higher BMI (P < 0.001). The prevalence of hypertension (P < 0.001), high FBS (P < 0.001), high cholesterol (P = 0.026), high triglyceride (P < 0.001) and high waist circumference (P < 0.001) was also significantly higher in patients with NAFLD. Taking all these together, patients with NAFLD had significantly higher prevalence of metabolic syndrome when compared to healthy subjects (P < 0.001).

**Table 3.** Comparison of the Socio-demographic, Anthropometric and Biochemical Characteristics Between Those With NAFLD and Healthy Subjects

Variable	Healthy (n=643)	NAFLD (n=176)	P value
**Age, y, mean ± SD**	41.5 ± 14.3	48.5 ± 11.4	< 0.001
**Sex, No. (%)**			0.004
Male	250 (38.9)	90 (51.1)	
Female	393 (61.1)	86 (48.9)	
**BMI, kg/m^2^, mean ± SD**	29.1 ± 4.2	25.4 ± 4.2	< 0.001
**Income**			0.619
Low (< 400 USD/month)	218 (33.9)	69 (39.2)	
Mid(400 – 1000 USD/month)	385 (59.9)	98 (55.7)	
High (≥ 1000 USD/ month)	40 (6.2)	9 (5.1)	
**Years of education, No. (%) **			0.903
Illiterate	38 (5.9)	15 (8.5)	
< Diploma	167 (25.9)	45 (25.5)	
Diploma	226 (34.9)	59 (33.5)	
Bachelor of Science (BS)	145 (22.5)	38 (21.6)	
Master of Science (MS)	33 (5.1)	10 (5.8)	
Doctorate	34 (5.7)	9 (5.1)	
**Hypertension, No. (%)**			< 0.001
Yes	108 (16.8)	60 (34.1)	
No	535 (83.2)	116 (65.9)	
**Fasting Blood glucose, mg/dL, No. (%)**			< 0.001
High	89 (13.8)	78 (44.3)	
Normal	554 (86.2)	98 (55.7)	
**Cholesterol, mg/dL, No. (%)**			0.026
High	61 (9.5)	27 (15.7)	
Normal	582 (90.5)	149 (84.3)	
**Triglyceride, mg/dL, No. (%)**			< 0.001
High	182 (28.3)	105 (59.7)	
Normal	461 (71.7)	71 (40.3)	
**LDL, mg/dL, No. (%)**			0.087
High	40 (6.2)	18 (10.2)	
Normal	603 (93.8)	158 (89.8)	
**HDL, mg/dL, No. (%)**			0.929
Low	237 (36.8)	64 (36.4)	
Normal	406 (63.2)	112 (63.6)	
**Waist Circumference, cm, No. (%)**			< 0.001
High	181 (28.1)	106 (60.2)	
Normal	462 (71.9)	70 (39.8)	
**Metabolic Syndrome, No. (%)**			< 0.001
Yes	166 (14.0)	76 (43.2)	
No	553 (86.0)	100 (56.8)	

Among the patients with NAFLD, there were 29 (16.4%) patients with normal BMI (< 25 kg/m^2^). Table 4 compares the characteristics of these patients with those having high BMI. NAFLD patient with high BMI had significantly higher levels of cholesterol (P = 0.038), waist circumference (P < 0.001) and higher prevalence of metabolic syndrome (P = 0.025) when compared to those with normal BMI.

 There was no significant difference between these two groups regarding socio-demographic characteristics including education (P = 0.836) and income (P = 0.782). Also, we did not observe a significant difference between overweight and non overweight patients with NAFLD regarding Hb (14.7 ± 1.6 vs. 15.1 ± 1.1 mg/dL; P = 0.328), serum ferritin (132.7 ± 61.8 vs. 118.7 ± 74.4 µg/dL; P = 0.269), gamma-glutamyltranspeptidase (44.3 ± 13.7 vs. 40.9±16.2 IU/L; P = 0.610) and serum albumin (4.5 ± 0.4 vs. 4.6 ± 0.5 mg/dL; P = 0.142).

**Table 4.** Comparison of Anthropometric and Biochemical Characteristics of NAFLD Patients with Normal BMI vs. High BMI

Variable	High BMI (n=147)	Normal BMI (n=29)	P value
**Age, y, mean ± SD**	48.3 ± 10.8	49.8 ± 13.9	0.517
**Sex** **, No. (%)**			
Male	71 (48.3)	19 (65.5)	0.106
Female	76 (51.7)	10 (34.5)	
**Hypertension**			0.998
Yes	50 (34.0)	10 (34.5)	
No	97 (66.0)	19 (65.5)	
**Fasting blood glucose, mg/dL, No. (%)**			0.152
High	69 (46.9)	9 (31.0)	
Normal	78 (53.1)	20 (69.0)	
**Cholesterol, mg/dL, No. (%)**			0.088
High	21 (14.3)	8 (27.6)	
Normal	126 (85.7)	21 (72.4)	
**Triglyceride, mg/dL, No. (%)**			0.038
High	93 (63.3)	12 (41.4)	
Normal	54 (36.7)	17 (58.6)	
**LDL, mg/dL, No. (%)**			0.176
High	15 (10.2)	5 (17.2)	
Normal	132 (89.8)	24 (82.8)	
**HDL, mg/dL, No. (%)**			0.531
Low	58 (39.4)	9 (31.0)	
Normal	89 (60.6)	20 (69.0)	
**Waist circumference, cm, No. (%)**			< 0.001
High	101 (68.7)	5 (17.2)	
Normal	46 (31.3)	24 (82.8)	
**Metabolic Syndrome, No. (%)**			0.025
Yes	69 (46.9)	7 (24.1)	
No	78 (53.1)	22 (75.9)	

## 5. Discussion

NAFLD is among the common chronic liver diseases with wide variety of factors including genetic, environmental, metabolic, and stress-related. The natural history of NAFLD ranges from asymptomatic indolent to the end stage liver disease. The prevalence of ultrasonographically diagnosed NAFLD in industrialized countries ranges from 20% to 60% (21) with 21.8% in Japan and 24.3% in South Korea (22, 23). Several studies have investigated the prevalence of NAFLD and NASH in Iranian population (13-16). Alavian et al. (13) reported a NAFLD prevalence of 7.1% in Iranian children while Sohrabpour et al. (14) reported a NASH prevalence of 2.9% in Iranian general population adults through a countrywide study which was in consistent with Rogha et al. (15) who reported a NASH prevalence of 3.3% in a sample of Iranian adults. The highest reported prevalence of NAFLD in Iranian adults was among the patients with type 2 DM which was as high as 55.8% (16). Our study showed that approximately 21.5% of Iranian adults had NAFLD, which is much higher than previous study of Iran (13-16) and eastern countries (8).

Although the variations in the prevalence of the NAFLD can be attributable to genetic and environmental background, the differences in methodology and diagnostic criteria for NAFLD are another major problem. We included a randomly selected sample of Iranian adult general population who underwent a routine health check-up. Ultrasonography was the basis of NAFLD diagnosis in our study. The prevalence of 21.5% ultrasonographically diagnosed NAFLD in this study was lower than Germany (40.0%) (24), Sri Lanka (32.6%) (25), USA (33%) (26) and Japan (21.8%) (22)but higher than Italy (20%) (27), Taiwan (11.5%) (28), China (12.5% and17.2%) (4, 8), Philippines (12.2%) (29) and Brazil (2.3%) (30). Most likely, this difference can be explained based on the higher prevalence of (components of) the metabolic syndrome in patients as compared to randomly selected individuals within the general population.We found that NAFLD was associated with age, BMI, hypertension, high FBS, high cholesterol, high triglyceride and high waist circumference. These determinants of NAFLD are the metabolic and anthropometric features of metabolic syndrome (9). Thus NAFLD is closely associated with metabolic syndrome in our region (southern Iran). Subjects with metabolic syndromes are at increased risk of developing diabetes mellitus and cardiovascular disease (10). Thus, NAFLD could be considered as an additional feature of metabolic syndrome.In this study, the prevalence of NAFLD was higher in males than in females. A similar finding has been noted in several previous studies (31, 32). These age-related gender differences may be related to reduced androsterone in males and low estrogen levels and relatively increased androsterone after menopause in females of more than 60 years old (23). This possibility implies that female hormones might have favorable effects on lipid metabolism in the liver. Vice versa, androsterone and androgens may have unfavorable effects on liver function and hepatocytes. Another explanation for high male to female ration in NAFLD could be the higher consumption of alcoholic beverages by men compared to women.It is well recognized that the pattern of obesity plays an important role in NAFLD development and progression (33). The critical pathophysiological step in the development of NAFLD is considered to be visceral obesity. This effect is independent of hepatic steatosis and insulin resistance. In addition to BMI and waist circumference, it has been demonstrated that subcutaneous fat thickness measured by ultrasound, is significantly correlated with ultrasound diagnosed NAFLD (24). This measure is feasible, easy to obtain,inexpensive and provides the clinician with quantitative values. Thus, it could be used in combination with visceral or perihepatic adipose tissue thickness in the diagnosis of NAFLD (34). It is clearly demonstrated that obesity (29, 35, 36), DM (16) and dyslipidemia (36) are associated with NAFLD. However, several studies have reported NAFLD in individuals lacking these risk factors, specially obesity (25, 35, 37). In addition, although Asians are less obese compared to westerns, the prevalence of NAFLD has not been found to be lower in these nations (8, 16, 23, 25, 28, 29, 31, 32, 35, 36). Kim and co-workers reported a prevalence of 23.4 for NAFLD in nondiabetic, nonobese adults which is comparable to several reports which determined the prevalence of NAFLD in general population (8, 23, 28). This could be explained by other undetermined factors such as genetic background as well as lifestyle. It is presumed that high carbohydrate intake would lead to the development and progression of NAFLD even in nondiabetic, nonobese adults (35, 38). It has also been reported that percentage body fat is an independent risk factor of NAFLD in nondiabetic, non overweight adults (35). In other words, non overweight individuals with excessive fat percentage are at higher risk of development of NAFLD. In this study, we demonstrated that overweight NAFLD patients had significantly higher levels of cholesterol, waist circumference and higher prevalence of metabolic syndrome when compared to non overweight patients. The risk factors and predictors of NAFLD in non overweight individuals should be investigated in more details in future.We have noted some limitations to this study. First, the study population was slightly low and future studies with more participants are recommended. However, the precise cluster sampling used in this study resulted in a study population which is representative of the whole community in our region. Second, the measurements and the clinical examination were performed by several physicians and nurses which has resulted in inevitable interobserver variation affecting the reliability of the clinical findings and measurements. Third, ultrasonogrphic diagnosis of NAFLD is questionable. Currently using magnetic resonance imaging and liver biopsy are more acceptable for the diagnosis of NAFLD. In conclusion, the prevalence of NAFLD in Iranian adult general population is 21.5%, which is roughly high. NAFLD in Iranian population is associated with male sex, old age, obesity, and other features of metabolic syndrome. As NAFLD has the possibility of progression toward end-stage liver disease and is associated with increased cardiovascular risk, appropriate action should be undertaken in our region for screening and control of this disease. Preventive strategies should also be pursued in our region.
